# Carriage rate of CPE, ESBL-producing coliforms and MRSA in community settings in Suriname

**DOI:** 10.1093/jacamr/dlag169

**Published:** 2026-08-10

**Authors:** Carlotte M Rozemond, Malti R Adhin, Whitney Williams, Sabine C De Greeff, Engeline Van Duijkeren, Stephen G Vreden, Francis T Mawie, Ed Ijzerman, Joost Hordijk, Jaap T Van Dissel

**Affiliations:** National Institute for Public Health and the Environment (RIVM), Centre for Infectious Disease Control, Antonie van Leeuwenhoeklaan 9, Bilthoven, The Netherlands; Department of Infectious Diseases, Leiden University Medical Centre, Albinusdreef 2, Leiden, The Netherlands; Faculty of Medical Sciences, Department of Biochemistry, Anton de Kom Universiteit van Suriname, Kernkampweg 5, Paramaribo, Suriname; N.V. MyLab, Carusostraat 1, Paramaribo, Suriname; National Institute for Public Health and the Environment (RIVM), Centre for Infectious Disease Control, Antonie van Leeuwenhoeklaan 9, Bilthoven, The Netherlands; National Institute for Public Health and the Environment (RIVM), Centre for Infectious Disease Control, Antonie van Leeuwenhoeklaan 9, Bilthoven, The Netherlands; Foundation for the Advancement of Scientific Research Suriname (SWOS), K.P. van Westenstraat 9, Paramaribo, Suriname; Department of Medical Microbiology, Academic Hospital Paramaribo, Flustraat, Paramaribo, Suriname; Department of Medical Microbiology, Academic Hospital Paramaribo, Flustraat, Paramaribo, Suriname; National Institute for Public Health and the Environment (RIVM), Centre for Infectious Disease Control, Antonie van Leeuwenhoeklaan 9, Bilthoven, The Netherlands; Department of Infectious Diseases, Leiden University Medical Centre, Albinusdreef 2, Leiden, The Netherlands

## Abstract

**Background:**

Colonization by antimicrobial-resistant bacteria can cause difficult-to-treat infections, yet their carriage prevalence in Suriname is unknown. This cross-sectional study assessed intestinal community carriage and the molecular epidemiology of pathogens of public health importance, including carbapenemase-producing Enterobacterales (CPE), ESBL-producing coliforms (ESBL-c) and MRSA.

**Methods:**

Faecal samples were collected from 310 non-hospitalized patients between October 2024 and February 2025 and analysed using selective culture and WGS.

**Results:**

The prevalence of ESBL carriage was 12.3% (95% CI: 8.4%–15.6%). Most ESBL/plasmid-mediated AmpC genes belonged to the *bla*_CTX-M-1_-group. Among the detected ESBL-c isolates, four distinct genetic clusters were identified: two clusters had identical core genomes, and two clusters had related ones (≤10 allelic differences). No CPE were detected. Two MRSA and one *Staphylococcus argenteus* (MRSArg) with *mecA* genes were identified.

**Conclusions:**

This study demonstrated frequent ESBL carriage in community-dwelling individuals attending ambulatory care in Suriname. Genomic clustering suggests transmission or shared sources. In contrast, carriage of CPE and MRSA was uncommon. These findings may help clinicians and policymakers to make informed decisions and develop targeted strategies to limit the spread of antimicrobial resistance and improve global public health.

## Background

Antimicrobial resistance (AMR) is a major and growing global health threat. Of particular concern are carbapenemase-producing Enterobacterales (CPE), ESBL-producing coliforms (ESBL-c) and MRSA.^1^ These bacteria can cause severe, difficult-to-treat infections, particularly in vulnerable patient groups, with a high burden in low-resource settings.^2^

While these resistant bacteria receive considerable attention in hospital settings, they are not confined to healthcare settings and may be carried asymptomatically by the community. Community carriage facilitates human-to-human transmission and increases the risk of subsequent infections.^3–5^ Understanding local carriage prevalence helps to estimate the size of the AMR reservoir in the population.

Although the occurrence of AMR has been studied in hospitals and livestock in Suriname, a tropical middle-income country on the northern coast of South America, the size and molecular composition of the community reservoir remain unknown. We therefore examined the prevalence and genomic diversity of intestinal carriage of CPE, ESBL-c and MRSA among community-dwelling individuals attending ambulatory care in Suriname.

## Methods

### Ethics

The Centre for Clinical Expertise at the Dutch National Institute for Public Health and the Environment (RIVM) reviewed the study and confirmed that ethical approval was not required under Dutch and European regulations (study number Z&O-749). Use of anonymized residual material was in accordance with the facility’s terms and conditions and communicated to all clients.

### Sample collection

In this cross-sectional study, human faecal samples were collected between October 2024 and February 2025. At least 300 non-hospitalized patients visiting health service laboratories at over 60 locations across Suriname were included as a proxy for community settings. These laboratories routinely analyse faecal samples for worm eggs, digestion, Salmonella/Shigella, *Helicobacter pylori* and occult blood and perform general faeces analysis. After routine diagnostics, residual samples were aliquoted, homogenized in trypticase soy broth with 20% glycerol and stored at −20°C until transport to the RIVM for further analysis.

### Microbiological analysis

Microbiological methodology followed previously described procedures by Rozemond et al.^6^ Briefly, faecal samples were either cultured directly or first enriched in selective or non-selective media before plating on selective agar to detect CPE, ESBL-c and MRSA. In addition, all faecal samples were cultured on Brilliance *E. coli*/Coliform Selective Agar (Oxoid) to verify *Escherichia coli* or coliform growth. From each plate with typical colonies for CPE and ESBL-c, one presumptive colony was selected and identified by MALDI-TOF. Presumptive MRSA colonies were confirmed as *Staphylococcus aureus* with *spa*-genes typed by PCR. All identified isolates underwent WGS.

### Genomic analysis

Illumina short-read sequences were assembled using the in-house JUNO-pipeline (https://github.com/RIVM-bioinformatics/juno-assembly). Assemblies were screened with AMRFinder (download 2025-04-25) and PlasmidFinder (download 2025-04-28), using 60% coverage and 90% and 95% identity cut-offs, respectively. Core-genome multi-locus sequence typing (cgMLST) was performed using species-specific cgMLST schemes in CLC Genomics Workbench (version 25.0.1). For *Escherichia* spp., a scheme with 2513 core genes was used (update 2024-11-11); the *Klebsiella pneumoniae* species complex scheme included 629 core genes (update 2025-09-28), and the *S. aureus* scheme comprised 1716 core genes (update 2025-09-16). For Enterobacterales, relatedness was defined as ≤10 allelic differences (AD).^7^ For *S. aureus*, isolates with 0–8 AD were considered related, 9–29 AD possibly related and ≥30 AD unrelated.^8^

### Statistical

Descriptive statistics, proportions and confidence intervals were used to summarize the prevalence of CPE, ESBL-c and MRSA. Associations between age category, gender and ESBL carriage were assessed using Fisher’s exact test (with Monte Carlo simulation for age categories).

## Results

**Figure 1.**
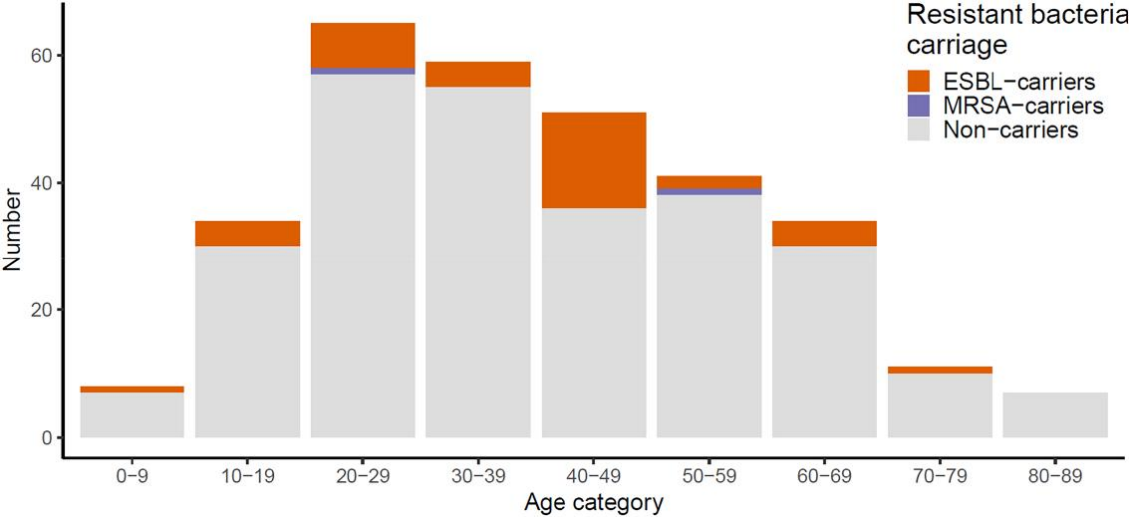
The distribution of ESBL-, MRSA- and non-carriers over age.

Faecal samples were collected from 310 non-hospitalized individuals with a median age of 38 years (range: 1–86; Figure 1). Samples were submitted for worm eggs (*n* = 151), digestion (*n* = 15), Salmonella/Shigella (*n* = 25), *H. pylori* (*n* = 31), occult blood (*n* = 78) and general faeces (*n* = 10) analysis. No bacterial growth was observed in 14 samples. The prevalence of ESBL-c carriage was 12.3% (38 out of 310 individuals) (95% CI: 8.4–15.6%), with the highest proportion of carriers in the 40–49-year age group (29%; Figure 1). The carriage rate ranged from 0% for general faeces cultures, 8% for Salmonella/Shigella, to 13% each for samples sent for *H. pylori*, occult blood, digestion and worm egg testing. Although most participants were female (59%), the proportion of ESBL carriers did not differ significantly between males and females. No resistant bacteria were found among the seven participants aged ≥80 years.

ESBL-producing *E. coli* were detected in 26 individuals (68% of ESBL-c positive samples; *n* = 38). ESBL-producing *K. pneumoniae* were detected in nine individuals (24%), and three ESBL-producing Enterobacter spp. (*Enterobacter cloacae*, *Enterobacter roggenkampii* and *Enterobacter hormaechei*) were detected in three individuals (8%). Most ESBL-c isolates (66%) were recovered after enrichment. No CPE was detected in the faecal samples. Three organisms of the genus *Staphylococcus* were identified: two MRSA isolates and one *Staphylococcus argenteus* isolate, resulting in an intestinal MRSA detection rate of 0.6% (95% CI: 0–1.5%).

A wide variety in STs was observed among ESBL-c. ST73 (*n* = 4), ST131 (*n* = 3), ST6448 (*n* = 2) and ST10955 (*n* = 2) were most frequently detected in *E. coli*. The other *E. coli* STs were distributed across 13 other STs. Among ESBL-producing *K. pneumoniae*, ESBL-producing Enterobacter spp. and MRSA isolates, no dominant STs were identified. *bla*_CTX-M-15_ was the most frequently detected ESBL gene (*n* = 8), followed by *bla*_CTX-M-27_, *bla*_CTX-M-3_ and *bla*_CTX-M-55_ (each *n* = 4). The three Enterobacter spp. isolates carried different AmpC genes: *bla*_CMH_, *bla*_MIR-9_ and *bla*_ACT-17_. Both MRSA isolates and the *S. argenteus* (MRSArg) isolate carried the *mecA* gene and tested negative for *pvl*.

**Figure 2.**
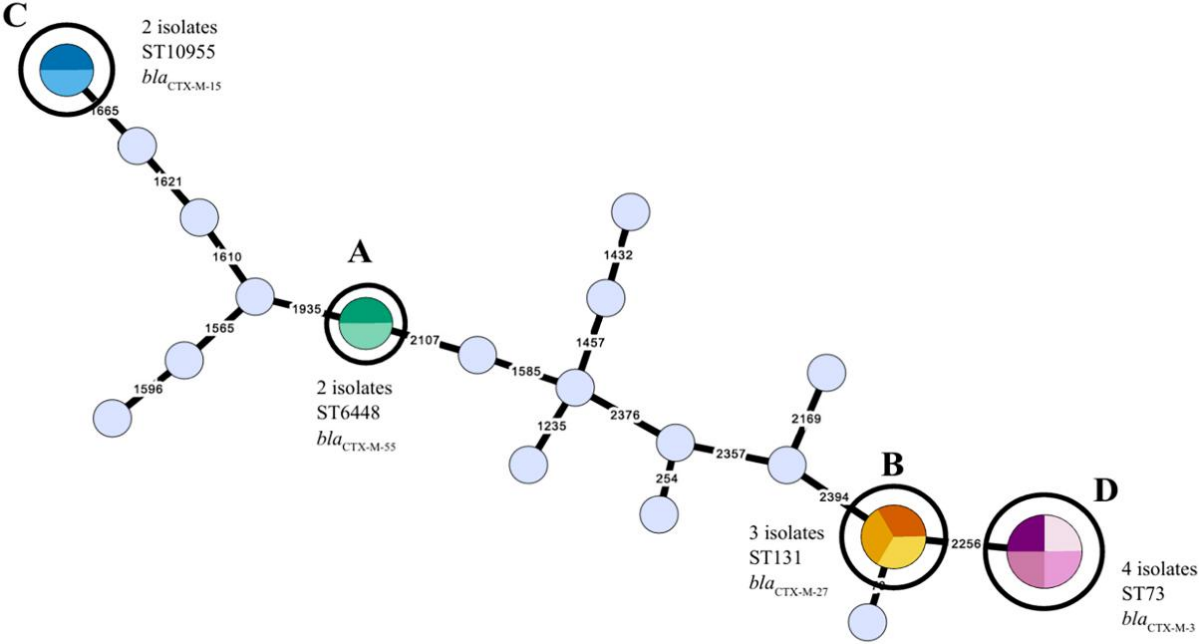
Cluster analysis for ESBL-producing *E. coli* collected from community settings in Suriname. Four clusters (≤10 AD) were indicated by circles. The figure was created using CLC Genomics Workbench (version 15.0.1) and finalised in Illustrator (version 29.8.2).

Based on core-genome analysis, four clusters were identified among the ESBL-producing *E. coli* isolates (Figure 2). One cluster consisted of two ST6448 isolates carrying *bla*_CTX-M-55_, which differed by only two AD (Figure 2a). Another cluster comprised three ST131 isolates harbouring *bla*_CTX-M-27,_ with four and nine AD (Figure 2b). Additionally, two clusters contained isolates with identical core genomes: two ST10955 carrying *bla*_CTX-M-15_ and four ST73 isolates carrying *bla*_CTX-M-3_ (0 AD) (Figure 2c and d). The two MRSA isolates showed >30 AD, and the nine ESBL-producing *K. pneumoniae* isolates showed >10 AD. These MRSA and ESBL-producing *K. pneumoniae* isolates were considered unrelated.

## Discussion

Our findings establish the first genomic baseline for community carriage of antimicrobial-resistant bacteria in Suriname. The presence of multiple cgMLST clusters among ambulatory individuals indicates circulation of successful ESBL strains outside hospital settings, although shared environmental exposure cannot be excluded. In contrast, CPE and MRSA carriage was uncommon.

Globally, ESBL-producing *E. coli* prevalence in community settings is estimated at 17.6%, with regional differences ranging from 6.0% in Europe to 35.1% in Southeast Asia and 10.3% in the Americas.^9^ Studies from open community settings have reported ESBL-Enterobacterales/*E. coli* prevalences of 8.3% in Brazil, 22.1% in Chile, 41.9% in Guatemala and 10.1% in a similar study population in the Netherlands.^10–13^ Our findings fall within the lower range of regional observations and are comparable to Dutch findings.

Regarding the molecular characteristics of ESBLs, we predominantly found *bla*_CTX-M-15_, like in the Netherlands. In contrast, *bla*_CTX-M-1_ was most prevalent in French Guiana, *bla*_CTX-M-55_ in Ecuador, *bla*_CTX-M-8_ in Brazil and *bla*_CTX-M-2_ in Peru and Bolivia.^11–14^ The predominance is likely influenced by the ESBL genes and sequence types circulating in local healthcare settings, livestock, food sources and the environment, and vice versa. Additionally, the observed genomic clusters may reflect shared sources or transmission events between individuals; however, due to the lack of epidemiological data, these links remain inconclusive.

Intestinal carriage of MRSA is estimated at around half the level of nasal carriage.^15^ Extrapolating this, the estimated prevalence of nasal MRSA carriage in our population would be approximately 1.2%, consistent with reported prevalences in the region: 0.7% in remote Brazilian communities, 2.3% in both a Brazilian city population and abattoir workers from Trinidad and Tobago, and 2.8% in Haiti.^16–19^ Although our study population mainly convened individuals with gastrointestinal complaints, it is unlikely that this has led to an overestimation of MRSA carriage prevalence, as gastrointestinal symptoms are generally not associated with MRSA colonization.^20,21^ This suggests that MRSA carriage in our study population is low and similar to that in the wider region.

Our study has several limitations. First, our study population consisted of individuals who visited ambulatory care, including those with gastrointestinal complaints. This pragmatic selection may have introduced selection bias, limiting generalizability to the general population. Secondly, the samples were not cultured before storage and transport at −20°C, which could have decreased bacterial viability and underestimated the presence. Lastly, we had limited information on patient characteristics, such as recent antimicrobial use or risk factors for AMR colonization (e.g. travel history, antibiotic use, recent hospitalization or occupation). This limited our ability to assess potential determinants for acquisition or dissemination of resistant bacteria. Future studies incorporating these factors would support the identification of specific risk groups and guide targeted interventions to reduce the spread of AMR within Suriname.

In conclusion, this study provides the first genomic inventory of clinically significant antimicrobial-resistant bacteria in community-dwelling individuals in Suriname. While community carriage cannot be entirely prevented, it contributes to spread and may hinder efforts to control AMR in healthcare settings. These findings provide an important baseline for future AMR surveillance and could help clinicians and policymakers in devising control strategies in Suriname.

## Data Availability

Sequence data are deposited in the European Nucleotide Archive (ENA) in project PRJEB110040.
